# A microfluidic approach to studying the injection flow of concentrated albumin solutions

**DOI:** 10.1007/s42452-021-04767-2

**Published:** 2021-08-24

**Authors:** Alfredo Lanzaro

**Affiliations:** 1 Present Address: Institute for Systems Rheology, Guangzhou University, Guangzhou, 510006 People’s Republic of China; 2 School of Chemical Engineering and Analytical Science, Faculty of Engineering and Physical Sciences, University of Manchester, Manchester, M13 9PL UK

**Keywords:** Biopharmaceuticals, Concentrated protein solutions, Microfluidics, Complex flows, Extensional rheometry

## Abstract

**Abstract:**

Subcutaneous injection by means of prefilled syringes allows patients to self-administrate high-concentration (100 g/L or more) protein-based drugs. Although the shear flow of concentrated globulins or monoclonal antibodies has been intensively studied and related to the injection force proper of SC processes, very small attention has been paid to the extensional behavior of this category of complex fluids. This work focuses on the flow of concentrated bovine serum albumin (BSA) solutions through a microfluidic “syringe-on-chip” contraction device which shares some similarities with the geometry of syringes used in SC self-injection. By comparing the velocity and pressure measurements in complex flow with rheometric shear measurements obtained by means of the “Rheo-chip” device, it is shown that the extensional viscosity plays an important role in the injection process of protinaceous drugs.

**Article Highlights:**

- A microfluidic “syringe on chip” device mimicking the injection flow of protinaceous drugs has been developed.
- The velocity field of concentrated BSA solutions through the “syringe on chip” is Newtonian-like.
- The extensional viscosity of concentrated protein solutions should also be considered when computing injection forces through needles.

## Introduction

Macromolecules such as monoclonal antibodies (mAbs) are increasingly being used for treating a large variety of diseases, including immunodeficiency, leukemia and arthritis [17], while globular proteins such as recombinant serum albumin are often employed as drug stabilizers [28, 59]. Subcutaneous (SC) injection by means of prefilled syringes coupled with autoinjector devices constitutes a viable alternative to intravenous delivery, as it allows the patients to self-administrate the protinaceous drug at home. Perceived pain and back pressure typically limit the highest deliverable volume to 1.5 mL [37, 58, 62]; therefore, biopharmaceuticals need to be concentrated above 100 g/L in order for the drug to be effective. Such large values of protein concentrations typically translate into large solution viscosity (up to 1 Pa s [2, 63]) as well as into shear thinning behavior [8, 11, 18, 68] at the shear rates typical of SC injection ($10^{4} \le {\dot{\gamma }} \le 10^{5}$ s$^{-1}$ for 27-G needle syringes, see [1]).

Several previous works have produced a calculation of the injection force of concentrated mAbs through syringes by assuming either Newtonian or shear-thinning viscosity [1, 7, 48]. It must however be observed that the flow of protein solutions through needles can be considered as a complex flow, because it contains not only a shear component, but also a well-defined extensional contribution which is localized in the contraction part between the syringe barrel and the needle. Thus, in order to develop a complete understanding of the origin of the forces arising when a biopharmaceutical drug is delivered by means of SC injection, one must characterize not only the shear, but also the extensional viscosity of concentrated protein solutions. While there is a large amount of works which focus on bulk shear viscosity [29, 36, 53, 68], high-frequency viscoelasticity [42, 43, 65] or interfacial shear rheometry [26, 57] of dense protein solutions, very little attention has so far been paid to the elongational behavior of this category of complex fluids.

The extensional viscosity of complex fluids is directly related to the amount of deformation underwent by macromolecules under flow. Following de Gennes’ work [10], a polymer in extensional flow undergoes a transition from coiled to uncoiled state if it is deformed with a strain rate whose magnitude is comparable to the inverse of the longest polymer relaxation time, and for a long enough time such that a sufficient amount of strain can be accumulated. For example, $\lambda$-bacteriophage DNA has a relaxation time of approximately 0.1 s [45], and hence it reaches the uncoiled state if extensional rates of 10 s$^{-1}$  or above are applied. While there is a general consensus in the literature on the flow conditions that need to be achieved in order to stretch DNA and linear polymer chains, the data related to proteins are more controversial. Jaspe and Hagen [27] estimated that horse cytochrome unravels under purely extensional flow at strain rates of $10^{7}$ s$^{-1}$  or above. Churchich et al. [9] suggest that also the relaxation time of a much larger globulin such as bovine serum albumin (BSA) is on the order of $10^{-7}$ s. This is in contrast to the experimental work of Bekard et al. [5], which suggest that bovine serum albumin (BSA) unfolds in Couette flows at shear rates as low as $10^{2}$ s$^{-1}$. By means of a microfluidic device, Schneider [54] demonstrated that the von Willebrand factor, a protein commonly found in human blood, can unravel at shear rates of about $10^{3}$ s$^{-1}$. Computer simulations [60, 61] showed that the unraveling under shear flow of ubiquitin and integrin gives rise to many more intermediates than in the case of homopolymers. The dynamics of unraveling also depend on whether the protein is anchored or not, and on which specific protein site is anchored. More recently, Dobson et al. [13] showed that BSA and monoclonal antibodies can also aggregate when they undergo multi-pass extensional flow at strain rates ${\dot{\varepsilon }} \cong 10^{4}$ s$^{-1}$. The authors suggest that the aggregation of protein molecules at large extensional rates is caused by flow-driven protein unfolding, which is in turn related to strong hydrodynamic forces.

The advent of microfluidics has made it possible to study flow regimes which could not be previously investigated. Due to characteristic sizes of just a few microns, microfabricated devices allow for fluids to be tested under very large (${\dot{\gamma }} \ge 10^{4}$ s$^{-1}$) rates of deformation, while Reynolds numbers (hence inertial effects) are almost negligible. When macromolecular solutions are subjected to large deformation rates in microscopic flows through abrupt [30, 35, 49, 50] and hyperbolic contractions [32] or “cross-slots” [3, 24, 33], nonlinear flow effects such as asymmetries in the flow field and vortex development are often witnessed. While a considerable amount of effort has been spent in studying microscopic flows of macromolecular fluids such as polymers, DNA and other biopolymers [19–21, 23], or wormlike micellar solutions [22, 44, 69], very little attention has so far been paid to the behavior of concentrated protein solutions at high deformation rate, complex flow conditions which mimic practical processes. It is then natural to ask if the flow-induced protein unraveling and/or aggregation phenomena discussed above can lead to nonlinear flow effects similar to what has been previously observed for other types of macromolecules.

In the present work, the commonly made assumption that the injection force of protinaceous drugs is made only by a purely shear contribution is critically assessed. This is done by means of a microfluidic platform which mimics the flow behavior of concentrated protein solutions when flowing through syringes. Three BSA solutions in a pH $=7.0$ buffer condition and at $c_{2}=100, 200$ and 300 g/L were used here. Such concentrations are above the physiological value ($7\times 10^{-4}$ M, equivalent to $c_{2} \approx 50$ g/L) typically found in human blood [14]. Nevertheless, BSA solutions at this pH and over this range of $c_{2}$  are expected to have a similar shear and extensional viscosity with respect to that of mAbs-based biopharmaceuticals [53]. The microscopic geometries employed here are straight channel and a “syringe-on-chip” device consisting in several sudden planar contractions with same contraction ratio as those found in 26-G, 27-G and 30-G syringe needles. From the first geometry, the steady shear viscosity $\eta \left( {\dot{\gamma }}\right)$  corresponding to a range of deformation rates ($10\, s^{-1} \le {\dot{\gamma }} \le 10^{4}\,\mathrm{s}^{-1}$) comparable with that of the syringe injection process is extracted. From the “syringe-on-chip” geometry, the velocity and local strain rate fields within a similar range of nominal deformation rates are measured by means of particle-image velocimetry (PIV) [39, 41], so that it is verified whether the flow of the BSA solutions retains a Newtonian character. Finally, the extra pressure drops from the “syringe-on-chip” device are critically compared with the results obtained from the steady shear flow measurements, so that the relative importance of shear and extensional viscosity in the overall injection force can be assessed.

## Materials

BSA (Cat. no. A7906) was obtained from Sigma-Aldrich. The protein was initially dissolved at a concentration $c_{2}=300$ g/L in a pre-filtered buffer made by NaCl (145 mM), sodium octanoate (8 mM) and Tween 80 (0.05 g/L) at a pH $=7.0$ and let to dissolve for 72 h at 5 $^{\circ }$C. The initial BSA sample was filtered with a 0.22-microns filter (Anotop 10 filters, GE Healthcare, Piscataway, CA) to remove irreversible aggregates, and then fresh buffer was used for all subsequent dilutions up to the desired $c_{2}=100$, 200 or 300 g/L. The concentration of the protein samples was confirmed by a UV-1600PC spectrophotometer (VWR) operating at a wavelength of 280 nm. The small-angle X-ray scattering (SAXS) measurements performed by Sønderby et al. [59] on recombinant serum albumin, a globulin very similar to BSA, demonstrated that protein–protein interactions are repulsive under the chosen buffer conditions.

## Experimental methodology

### Rheo-chip platform

**Fig. 1:**
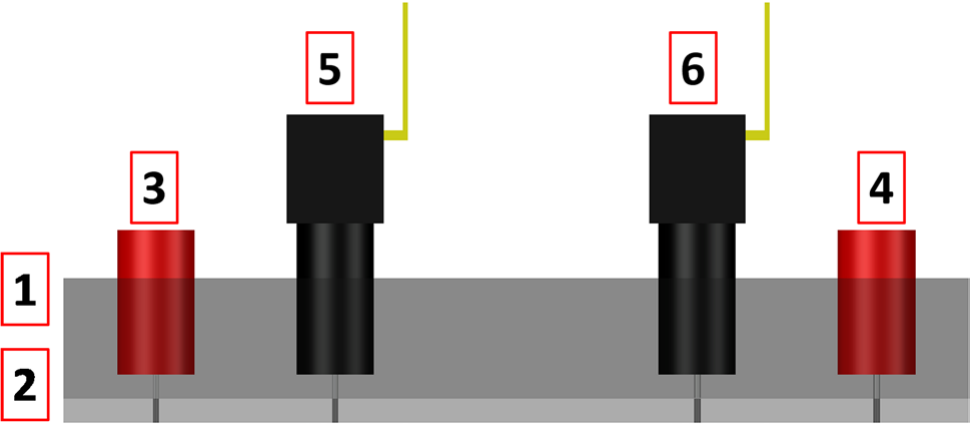
Schematic diagram of a “Rheo-chip” microdevice. *1* Delivery module. *2* Microfluidic flow geometry. *3* Inlet. *4* Outlet. *5* and *6* Honeywell pressure sensors

All microfluidic experiments presented in this work were run by means of the “Rheo-chip” platform [67]. It consists of a series of microchannels made in polymethilmetaacrilate (PMMA) by Epigem Ltd (Redcar, TS, UK). Each microchip features a delivery module where inlets, outlets and pressure taps are obtained. Pressure measurements were taken by means of Honeywell strain gauge sensors 26PCGFM6G (range 0–250 psi). The transient pressure signals were collected at 50 Hz by a cDAQ data acquisition system (National Instruments) and a LabVIEW software. The flow through microfluidic devices was controlled by means of a Nexus 6000 high force syringe pump (Chemyx Inc, Stafford, Tx) equipped with a 1-mL glass syringe (SGE Analytical, ID $=4.5$ mm). Note that, differently from other microfluidics-based rheometric techniques available elsewhere [46], the Rheo-chip technology employs replaceable pressure sensors, so that only the microfluidic chips need to be replaced if microchannels are clogged. This makes the rheometric characterisation of precious materials such as concentrated protein solutions much more cost-effective. The schematic diagrams of the “Rheo-chip” coupled with the pressure sensors are given in Fig. 1.

### Shear viscosity measurements

The steady shear viscosities of the model protein solutions were measured by means of Rheo-chips featuring a straight channel with width $w_{c}=800$ $\upmu$m, depth $h_{\text {nominal}}=50$ $\upmu$m and length $L=30$ mm. Two pressure taps were located along the microchannel at a distance of $L=30$ mm from each other, far away from the inlet and outlet, to measure the overall pressure drop $\varDelta P_{s}$. The pressure signals were collected by imposing an initial flow rate Q$_{\text {max}}$=10 mL/h and then waiting for the signal to reach a steady-state value. The flow rate was then reduced to 8 mL/h, and the procedure was repeated until a steady-state pressure drop corresponding to a flow rate $Q_{\text {min}}=0.25$ mL/h was measured [46]. For all samples studied in this paper, the nominal shear rate at walls is defined as

1 $$\begin{aligned} {\dot{\gamma }}_{N}=\frac{2Q}{h_{\text {true}}^{2}w_{c}}, \end{aligned}$$

where $h_{\text {true}}$  is found by fitting the steady-state pressure drop measurements of DI water with the analytical prediction for Newtonian flows through rectangular ducts given by White [64]. The corresponding shear viscosity is

2 $$\begin{aligned} \eta _{N}=\eta _{\text {buffer}}^{.}K\frac{\varDelta P_{s}\left( {\dot{\gamma }}\right) }{{\dot{\gamma }}}, \end{aligned}$$

where the calibration coefficient $\frac{1}{K}=\overline{\frac{\varDelta P_{\text {buffer}}}{{\dot{\gamma }}}}$  is obtained by averaging the ratios between steady-state pressure drops of buffer and the corresponding shear rates, and $\eta _{\text {buffer}}\cong$ 1 mPa s is the shear viscosity of buffer as measured by a TA ARES-G2 rheometer equipped with cone and plate measurement tool. The overall measurement time for each sample was 20 mins, approximately, and an amount of sample between 0.6 and 1 mL was used for each measurement. The impact of viscous heating on the rheometric measurements was estimated by the Nahme–Griffith number

3 $$\begin{aligned} Na= \frac{\eta _{0}\beta d_{h}^{2}{\dot{\gamma }}^{2}}{\kappa T}, \end{aligned}$$

where $\kappa \approx$0.5918 W/(m K) is the thermal conductivity of buffer and $\beta =\frac{T}{\eta _\text {buffer}}\left| \frac{d\eta }{dT}\right| _{T=T_{0}} \approx$ 2.6 is the logarithmic derivative of shear viscosity of buffer with temperature *T* evaluated at *T*=*T*$_{0}=25$ $^{\circ }$C. It expresses the ratio between the heat generated by viscous heating of samples and the heat released by thermal diffusion at the surface of the microchannels [46]. *Na* resulted to be $\le 10^{-5}$  for all the samples and shear rates studied here, which systematically excludes the influence of thermal effects.

### “Syringe-on-chip” microdevices

**Table 1:** The details of “syringe-on-chip” flow geometries

Flow cell	$w_{u}$ $[\upmu m]$	$w_{d}$ $[\upmu m]$	*h* $[\upmu m]$	$\alpha$	$\beta$	$\varepsilon _{H}$
26G	800	47	50	20	17.02	2.83
27G	800	38	50	20	21.05	3.05

$\alpha$  and $\beta$  denote the upstream aspect ratio and contraction ratio, respectively. $\varepsilon _{H}= \ln \beta$  is the Hencky strain

**Fig. 2:**
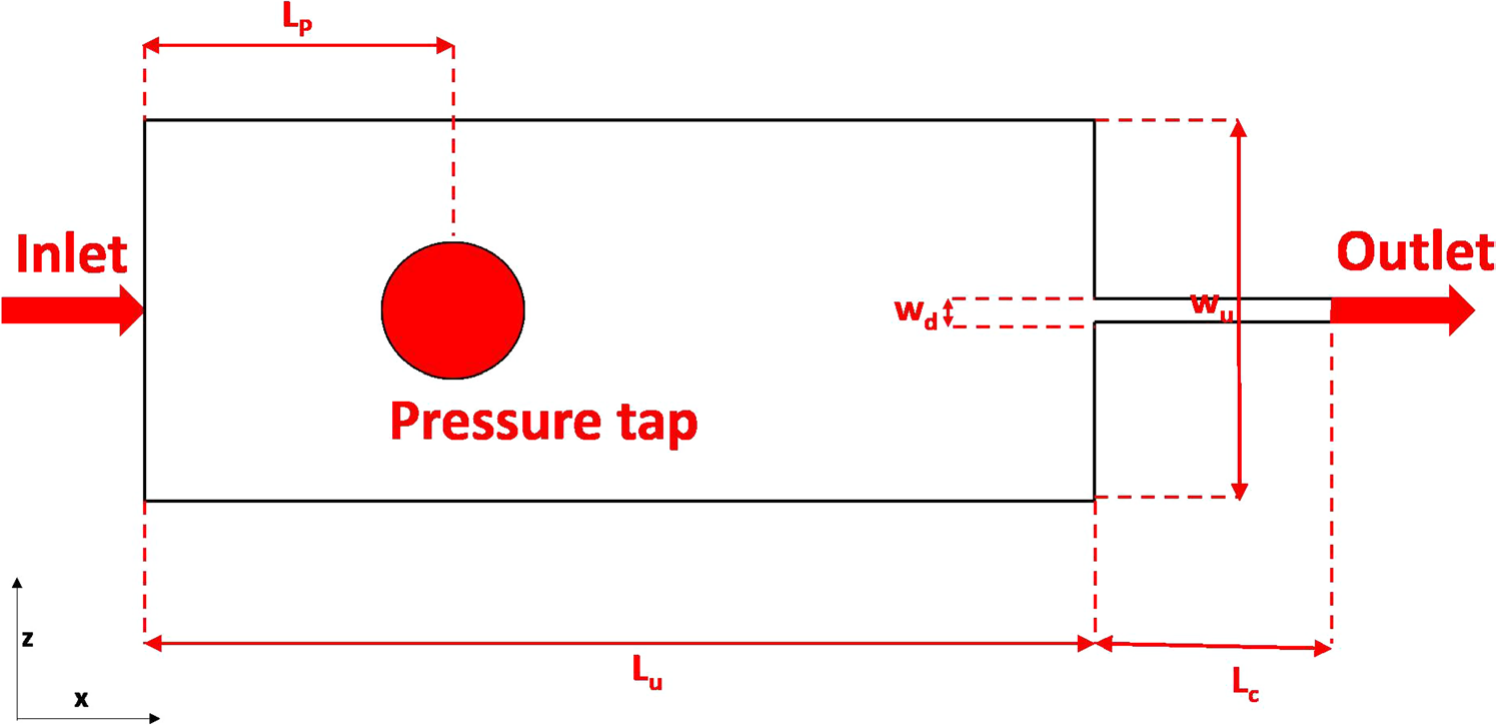
Schematic diagram of the “syringe-on-chip” microfluidic geometry. The dimensions $w_{u}$  and $w_{d}$  are given in Table 1, and $L_{p}$=14 mm, $L_{u}=32$ mm and $L_{c}=12$ mm

The two “syringe-on-chip” microdevices that are used in this work are presented in Fig. 2. They consist of planar sudden contraction devices with an upstream width $w_{u}=800$ $\upmu$m, channel depth $h=50$ $\upmu$m and downstream widths $w_{d}=47$ and 38 $\upmu$m. The corresponding contraction ratios $\beta$=17.02 and 21.05 are the same as what is typically found in syringes where the plunger diameter is 4.5 mm and the needle is a 26G or 27G [66]. Hence, from here onwards such microcontractions will be labeled as “26G” and “27G” geometry, respectively. Note that, due to fact that real syringes have axisymmetric instead of planar abrupt geometries, the flow through the syringe-on-chip devices feature lower Hencky strains $\varepsilon _{H}=\ln \beta$  compared to real syringes case, where $\varepsilon _{H}=2\ln \beta$. Each flow geometry contains a slot for a pressure sensor as in the figure. The outlet of each microchannel is connected to a fluid reservoir by means of a stainless steel tube with length $L_{\text {tube}}=100$ mm and inner diameter ID $=1/16$ inches (Cat. No. 56720-U, Sigma-Aldrich). The total pressure drop $\varDelta P_{\text {total}}$  measured by the sensor is related to the pressure drop $\varDelta P$  across the device by $\varDelta P_{\text {total}}=\varDelta P + \varDelta P_{\text {tube}}$, where $\varDelta P_{\text {tube}}=\frac{128\mu LQ}{\pi ID^{4}}$  is estimated by means of the Hagen–Poiseuille law. The $\varDelta P_{\text {tube}}$  contribution resulted to be 5$\%$  or less of the total pressure drop. The details of the flow geometries are given in Table 1. The flow of the concentrated BSA solutions in complex geometry is characterized in terms of the Reynolds number *Re*

4 $$\begin{aligned} \hbox {Re}=\frac{\rho <v_{d}>D_{h}}{\eta _{0}}, \end{aligned}$$

where $<v_{d}>$, $\rho \approx 1000$ g/L and $\eta _{0}$  are the average speed in the downstream channel, the fluid density and zero-shear viscosity, respectively, and $D_{h}=\frac{2hw_{d}}{h+w_{d}}$  is the hydraulic diameter of the downstream channel. In addition to Re, the Péclet number

5 $$\begin{aligned} \hbox {Pe}=\frac{\tau _{D}}{\tau _{F}} \end{aligned}$$

expresses the ratio between the characteristic timescales of flow, $\tau _{F}=\frac{1}{{\dot{\gamma }}}=\frac{w_{d}}{2<v_{d}>}$  and Brownian diffusion [34]. The diffusion time is $\tau _{D}=\frac{d_{h}^{2}}{D^{S}_{L}}$, where d$_{h}=7$ nm is the hydrodynamic diameter of BSA [4], and $D^{S}_{L}$  is the longtime self-diffusion coefficient of the BSA molecule, which is estimated by the generalized Stokes–Einstein relationship [51] as

6 $$\begin{aligned} D^{S}_{L}=\frac{k_{B}T}{3\pi \eta _{0}\eta d_{h}}, \end{aligned}$$

with $k_{B}$  and $T\approx$ 25 $^{\circ }$C being the Boltzmann constant and temperature, respectively.

### Particle-image velocimetry and data analysis method

**Fig. 3:**
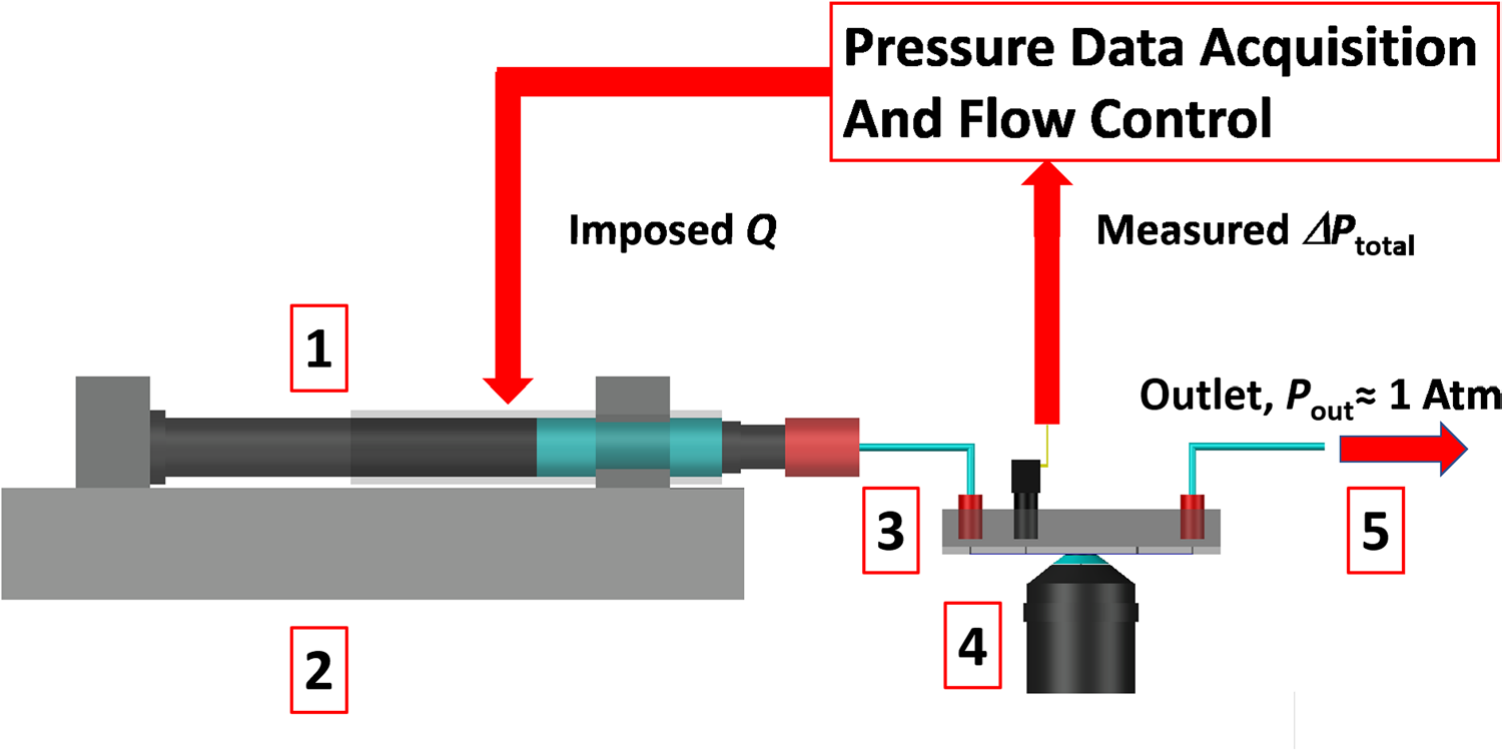
Schematic diagram of a typical “syringe-on-chip” experimental setup. The LabVIEW-based data acquisition platform allows to impose a flow rate *Q* to the syringe pump, and to measure the total pressure drop $\varDelta P_{\text {total}}$  from the pressure sensor. *1* Sample-filled glass syringe. *2* Syringe pump. *3* “Syringe-on-chip” microdevice. *4* Magnification lens. *5* Outlet piping

Velocity fields at the center plane of the “syringe-on-chip” flow channels were measured by a $\mu$-PIV system (TSI Instruments Ltd, USA). It consists of a pulsed Nd:YAG laser emitting at a wavelength of 532 nm, a Nikon TE2000-E fluorescent microscope, and an 8 Hz CCD camera with a resolution of 1280 $\times$ 1024 $\times$ 12 bits. A Nikon objective lens with magnification $M=10X$ and numerical aperture $NA=0.3$ was used. In Fig. 3, a schematic diagram of the “syringe-on-chip” device coupled with the PIV system is shown.

The BSA solutions were seeded with 0.01 wt% epi-fluorescent particles (diameter $d_{p}=1.0\,\upmu$m, $Ex_{\text {max}}/Em_{\text {max}}=542/612$, Duke Scientific Co.). For the above setup, the estimated measurement depth is 26 $\upmu$m, equivalent to 52%  of the channel depth [38]. All velocity measurements were taken after the pressure drop reached a steady value. Velocity fields were obtained by means of a Nyquist grid engine and a cross-correlation PIV algorithm supplied by TSI. All the $\mu$-PIV measurements presented in this work were performed at the middleplane of flow channel, determined by positioning the focus plane at half of the depth between the top and bottom surfaces of the microchannel, using a high-precision y-axis linear motor with step resolution 50 nm. The error in the velocity measurements is about 12–16% below the true value. This systematic error could be reduced where a higher magnification objective lens with a larger *NA* used. The details of the $\mu$-PIV techniques can be found in [39, 52]. All the velocity fields presented in this work were obtained by averaging 50 consecutive instantaneous velocity fields, corresponding to an overall measurement time of 12.5 s. From the measured two-dimensional planar velocity field ${\underline{v}}= {\underline{v}}(x,z)$, the largest eigenvalue $\lambda _{1}$ of the strain rate tensor $\underline{{\underline{D}}}=\frac{1}{2}\left( \nabla {\underline{v}}+\nabla {\underline{v}}^{T}\right)$  is then evaluated to construct the local extensional rate field ${\dot{\varepsilon }}\left( x,z\right)$. The uncertainty about the PIV velocity measurements is about 12–16%. More information on the computation of ${\dot{\varepsilon }}_{1}\left( x,z\right)$  can be found in [33].

## Results

### BSA solutions under steady shear flow

**Fig. 4:**
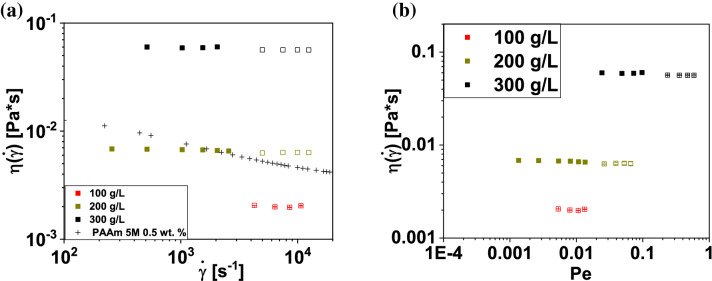
Shear viscosities plotted vs shear rate (**a**)  and vs the Péclet number (**b**) for the 100, 200 and 300 g/L BSA solutions. Filled and empty symbols indicate measurements taken with the 800 and 200 $\upmu$m Rheo-chip, respectively. In **a**, the shear viscosity of a 0.5 wt% solution of 5M polyacrylamide is also shown for a comparison [30]

The shear viscosity of the BSA solutions is plotted as a function of the shear rate 200 s$^{-1} \le {\dot{\gamma }} \le 2\times 10^{4}$ s$^{-1}$ in Fig. 4a. The viscosity curve of a weakly shear thinning, semi-dilute polyacrylamide (PAAm) solution [30] is also shown for a comparison. The rheograms of BSA appeared to be essentially flat, which is attributed to Pe being below unity throughout the imposed range of ${\dot{\gamma }}$  (see Fig. 4b). A recent work [12] demonstrated that concentrated lysozyme solutions at 5 $^{\circ }$C undergo shear thinning over a similar range of ${\dot{\gamma }}$  than what studied here. Such nonlinear effect was attributed to short-range attractive interactions, which lead to the formation of intermediate structures with a size larger than that of the monomer. The absence of shear thinning effect suggests that no intermediate structures are formed in the BSA solutions studied in this work. Unlike the BSA conditions studied here, mAb solutions are known to display shear-thinning effect even at $\mathrm{Pe}<<1$ [11, 18, 47]. In a recent work, the Rheo-chip technique adopted here has been used for studying the formulation dependence of $\eta _{0}$ of concentrated solutions of two mAbs [56]. Moreover, in a future work the relationship between the protein–protein interactions and the shear thinning behavior of concentrated solutions of the same two mAbs will be investigated in detail.

### Velocity and local extensional rate fields in “syringe on chip”

**Fig. 5:**
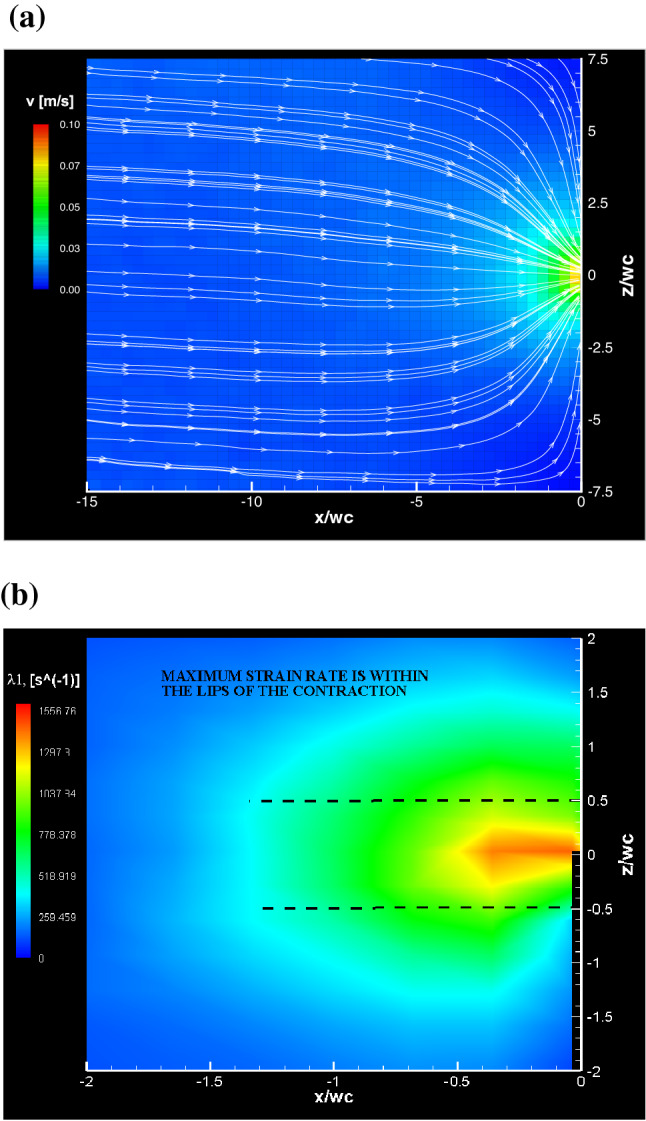
Velocity field (**a**)  and local strain field (**b**) for a Re $=0.09$, Pe $=0.24$ flow of a 300 g/L BSA solution through a “26-G” microchannel. The origin $(x,z)=(0,0)$ is located in the middle of the contraction throat

In Fig. 5a, the PIV-measured, planar velocity field of the Re $=0.09$, Pe $=0.24$ flow of the 300 g/L BSA solution through the 26-G microchannel is shown, to demonstrate its Newtonian-like behavior. To further assess the linearity of the flow field, the ${\dot{\varepsilon }}\left( x,z\right)$  field of the same BSA solution is plotted in Fig. 5b. The maximum measured extensional rate is localized near the contraction throat and resulted to be $\approx$ 1500 s$^{-1}$. Such strain rate is one order of magnitude above the threshold value for BSA unfolding ($10^{2}$  s$^{-1}$) reported by Bekard et al. [5], and about 10 times below the strain rate at which extensional flow-driven aggregation of BSA in multi-pass extensional flow occurs [13]. The maximum measured value of the extensional rate is located at the point $\left( x,z\right) \approx \left( 0,0\right)$. This is a further indication of the absence nonlinear flow effects, like steady viscoelastic flows or lip vortices [50], which are typically revealed by the maximum strain rate point being located at $x=0$  and $\left| \frac{z}{w_{d}}\right| >0.5$, that is, outside the lips of the contraction [6, 31, 32]. Therefore, these results indicate that the BSA molecules did not unfold under the existing flow conditions or, even if any unfolding event occurred, it was not released a sufficient amount of elastic energy to trigger the onset of non-Newtonian flow effects.

**Fig. 6:**
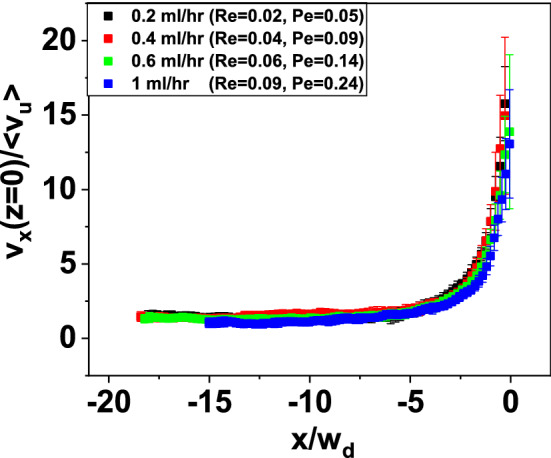
The non-dimensionalized axial velocity profiles along the $z=0$ line measured for the 300 g/L BSA solution through the “26-G” microchannel for 0.02 $\le \mathrm{Re} \le$  0.09, 0.05 $\le \mathrm{Pe} \le$  0.24

In Fig. 6, the dimensionless axial velocity profiles $\frac{v\left( x=0\right) }{<v_{u}>}$, where $<v_{u}>=\frac{Q}{hw_{u}}$  is the mean flow velocity in the upstream channel, are shown for 0.2 mL/h $\le Q \le$  1 mL/h. The velocity profiles overlap within the range of measured Re, which again indicates that the flow field is always Newtonian-like within the range of explored flow rates.

### Pressure measurements through “syringe on chip”

**Fig. 7:**
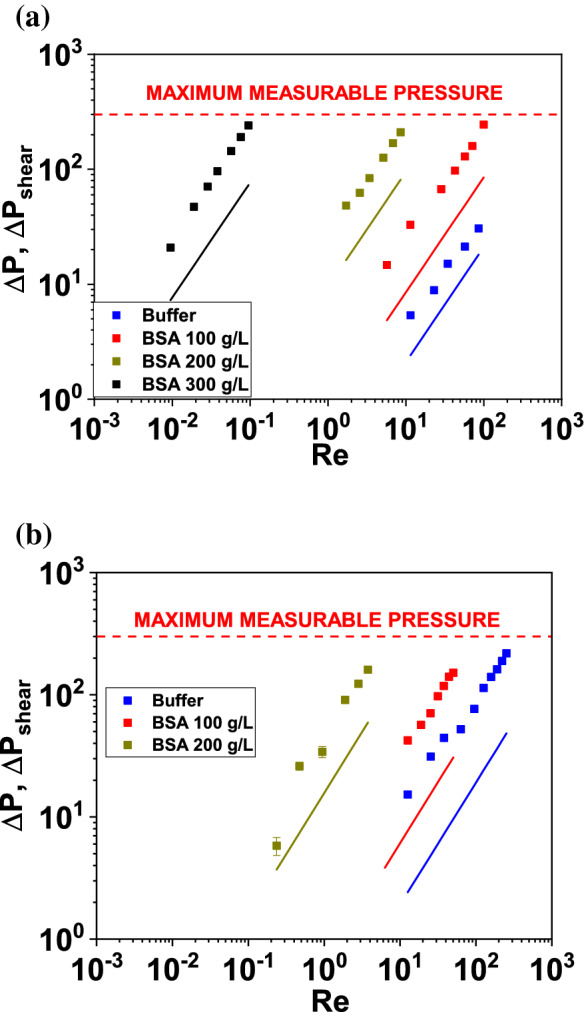
The steady pressure drops through the “26-G ” (**a**) and “27-G ” (**b**) channels plotted as a function of Re for buffer and the BSA solutions. Symbols refer to experimental data points, and lines correspond to the analytical prediction as in Eqs. (7) and (8)

**Fig. 8:**
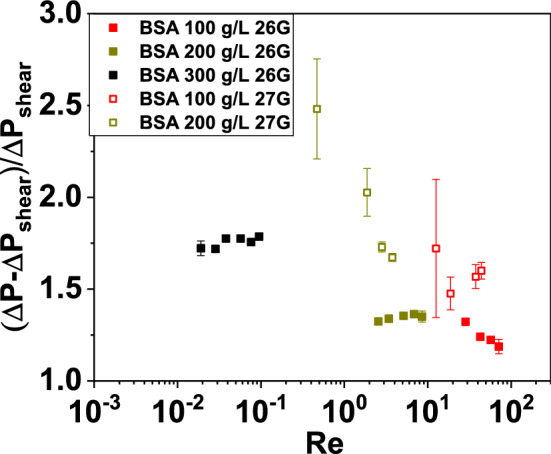
The non-dimensionalized extra pressure drop $\varDelta P'=\frac{\varDelta P-\varDelta P_{\text {shear}}}{\varDelta P_{\text {shear}}}$  plotted as a function of Re for the flow of the BSA solutions through the “26-G” and the “27-G” channels

In Fig. 7, the pressure measurements through the 26-G microcontraction are plotted as a function of Re for the buffer as well as for the 100 g/L and the 200 g/L BSA solutions. The experimental data are compared with the corresponding steady-state pressure drop contributions $\varDelta P_{\text {shear}}$, which are estimated as

7 $$\begin{aligned} \varDelta P_{\text {shear}}=L_{1}\frac{dP}{dz}\left( \alpha _{1}\right) +L_{2}\frac{dP}{dz}\left( \alpha _{2}\right) , \end{aligned}$$

where the pressure gradients $\frac{dP}{dz}\left( \alpha _{i}\right)$  are computed according to the analytical prediction for Newtonian flows through rectangular ducts given to White [64],

8 $$\begin{aligned} \frac{dP}{dz}\left( \alpha _{i}\right) =\frac{3\eta _{0}}{4w_{c}h^{3}\alpha _{i}^{4}}\frac{Q}{1-\frac{192\alpha _{i}}{\pi ^{5}K}}, \end{aligned}$$

where $\alpha _{1}=\frac{w_{u}}{h}$, $\alpha _{2}=\frac{w_{d}}{h}$, and $K=\Sigma _{n=1,3,5\ldots }^{+\infty }\frac{\tanh \left( \frac{n\pi }{2\alpha _{i}}\right) }{n^{5}}$. Such prediction ignores entry and exit effects and assumes that the flow instantaneously reaches the fully developed profile in both the upstream and downstream regions. The total pressure drop across the microchannels is above the purely shear contribution for all the BSA solutions. Such discrepancy is attributed to extensional effects across the sudden contraction, which are in turn related to the extensional viscosity of the model BSA solutions [21, 23, 33]. Due to the fact that the flow geometry used here is a sudden contraction, the difference between the total pressure drop and the purely shear contribution will be larger than in the case of Newtonian flows through hyperbolic contractions [41]. The extra pressure drop $\varDelta P'=\frac{\varDelta P-\varDelta P_{\text {shear}}}{\varDelta P_{\text {shear}}}$  for the BSA solutions across the 26G and 27G flow geometries resulted to be within the range $1.5 \le \varDelta P' \le 3$  throughout the explored range of Re (see Fig. 8). The $\varDelta P'$  of the more viscous 300 g/L solution through the 26-G channel resulted larger than that of the other two solutions. Moreover, the $\varDelta P'$  of the 100 and 200 g/L solutions through the 27-G channel are above the corresponding values for the 26-G channel because the Hencky strain is larger for the 27G than for the 26G geometry. Note that the simulations carried out by Oliveira et al. [40] for planar contraction-expansion flows of Newtonian fluids show that the Couette correction (analogous to the $\varDelta P'$  defined here) increases sharply with Re for Re > 1. This is due to the fact that, differently than in the work by Oliveira et al., the flow geometries adopted here do not include an expansion part, where inertia-driven flow instabilities, which also cost nonlinearity in the pressure drop-flow rate curve, are typically observed.

Previous works [1, 15] do not consider the contribution to pressure drop due to extensional forces [21, 23] in modeling the injection force of dense protein solutions through syringes. More specifically, the authors assume that the total injection force is composed by only two components: an hydrodynamic contribution, which is based on purely shear forces, and a friction force which takes into account the interaction between plunger and syringe barrel. On the other hand, the results presented here suggest that the overall injection force of biopharmaceuticals through syringes also contains a contribution due to purely extensional forces, which cannot be neglected in order for accurate predictions to be computed.

## Conclusions

Using the microfluidic Rheo-Chip platform, the flow of concentrated (100 g/L $\le c_{2} \le$  300 g/L) BSA solutions under repulsive conditions was studied through shear, and contraction flows mimicking the typical geometry of syringes equipped with 26-G and 27-G needles. The shear viscosity retained a Newtonian behavior across the range of investigated shear rates ($10\, \text {s}^{-1} \le {\dot{\gamma }}, \dot{\eta _{\text {ext.}}} \le 10^{4}\, \text {s}^{-1}$), which was attributed to the Péclet number being below unity. Similarly, the flow of the 300 g/L BSA solution through the 26-G “syringe-on-chip” microdevices appeared to be Newtonian-like up to Pe $=0.24$, which was likely because the BSA macromolecule retains its stability under the flow conditions imposed here [13].

Importantly, this work shows that the extensional viscosity of concentrated biopharmaceutical solutions also needs to be considered when one calculates the total force of syringe injection tests, because the measured pressure drop of the BSA solutions across the syringe-like microdevice resulted to be well above the purely shear contribution. This implies that the extensional viscosity plays an important role in determining the overall pressure drop of the complex flows underwent by protein solutions through needles, even if it is Newtonian. The extensional contributions are typically neglected in the formulation of predictions of injection forces [1, 7, 15, 48] and should then be considered if more realistic calculations have to be implemented.

Additionally, because the typical shear rate experienced by blood ranges from 10 $\text {s}^{-1}$  in the ascending aorta [16] up to 100 $\text {s}^{-1}$  in capillaries [25], while its pH $\approx$ 7.4 [55] is close to what probed here. Therefore, this work also indicates that human serum albumin maintains its native conformation under physiological flow conditions.
